# Detection and Phylogenetic Analysis of Group 1 Coronaviruses in South American Bats

**DOI:** 10.3201/eid1412.080642

**Published:** 2008-12

**Authors:** Christine V.F. Carrington, Jerome E. Foster, Hua Chen Zhu, Jin Xia Zhang, Gavin J.D. Smith, Nadin Thompson, Albert J. Auguste, Vernie Ramkissoon, Abiodun A. Adesiyun, Yi Guan

**Affiliations:** University of the West Indies, St. Augustine, Republic of Trinidad and Tobago (C.V.F. Carrington, J.E. Foster, N. Thompson, A.J. Auguste, V. Ramkissoon, A.A. Adesiyun); University of Hong Kong, Hong Kong Special Administrative Region, People’s Republic of China (H.C. Zhu, J.X. Zhang, G.J.D. Smith, Y. Guan)

**Keywords:** Bat, coronavirus, Bt-CoV, Trinidad, South America, dispatch

## Abstract

Bat coronaviruses (Bt-CoVs) are thought to be the precursors of severe acute respiratory syndrome coronavirus. We detected Bt-CoVs in 2 bat species from Trinidad. Phylogenetic analysis of the RNA-dependent RNA polymerase gene and helicase confirmed them as group 1 coronaviruses.

Bats are of particular interest as reservoirs for potentially emergent pathogens. Because of their abundance, wide distribution, and mobility, bats confer a greater risk for zoonotic transmission than other animals (*1*). Bats have long been known as the natural hosts for rabies virus and other lyssaviruses and were more recently identified as the reservoirs for emerging viruses such as Ebola, Hendra, and Nipah virus (reviewed in *1*). The search for the animal reservoir of the severe acute respiratory syndrome coronavirus (SARS-CoV) led to extensive surveys of coronaviruses in wild and domestic animal populations in China, resulting in the detection of a wide variety of novel bat coronaviruses (Bt-CoVs) (*2*–*5*). The data suggest that the progenitor of the SARS-CoV, and all other coronaviruses in other animal hosts, originated in bats (*4*). Recent reports by Dominguez et al. (*6*) and Gloza-Rausch et al*.* (*7*) confirmed the existence of Bt-CoVs outside China, in the United States and Germany, respectively. Additionally antibodies reactive with SARS-CoV have been detected in African bat species (*8*). We report the detection and characterization of CoVs in bats from Trinidad, the southernmost island of the Caribbean archipelago, located 9 km (5.5 miles) off the northeastern coast of South America.

## The Study

A total of 114 bats collected from their natural habitats from December 2006 through July 2007 (8 species from 10 locations; Table) were euthanized after being deeply anesthetized with 2% xylazine and 10% ketamine administered subcutaneously (in some cases after sedation with CO_2_). The bats were then taxonomically classified on the basis of morphology, and the carcasses were stored at –70°C until used. For sampling, the carcasses were thawed at 4°C for 3–4 hours, then oropharyngeal and anal samples were taken with Dacron-tipped swabs that were then placed in RNA*later* (Ambion, Austin, TX, USA) and stored at –20°C until used.

**Table:** Number and location of bat species collected and tested from December2006 through July 2007, with bat coronavirus species status

Species (family)	Location (no.)	Total no. tested (no. positive)
*Carollia perspicillata* (Phyllostomidae)	Arima (2), Fyzabad (2)*, Tabaquite (1)	5 (1)
*Glossophaga soricina* (Phyllostomidae)	Couva (12)*, Tabaquite (7)	21 (1)
*Noctilio leporinus* (Noctilionidae)	Couva (6)	6 (0)
*Desmodus rotundus* (Phyllostomidae)	Fyzabad (3), Morne Diablo (3), Rousillac (1), La Brae (7)	14 (0)
*Pteronotus parnelli* (Mormoopidae)	Tabaquite (29), Wallerfield (2)	31 (0)
*Molossus major* (Molossidae)	Talparo (25)	25 (0)
*Mormoops sp.* (Mormoopidae)	Tamana (1)	1 (0)
*Phyllostomus hastatus* (Phyllostomidae)	Wallerfield (11)	11 (0)
Total	10 locations; 8 species	114 (2)

*Indicates group from which coronavirus positive bats originated.

CoV detection and sequencing were conducted as previously described (*4*). Briefly, viral RNA was extracted from swabs by using the QIAamp viral RNA minikit (QIAGEN, Westburg, the Netherlands) and used as the template for reverse transcription–PCR (RT-PCR) detection of the CoV RNA–dependent RNA polymerase (RdRp) gene (*9*). Primers based on the RdRp gene, conserved for all known coronaviruses, were then used for RT-PCR detection. The RdRp PCR products were gel purified by using the QIAquick PCR purification kit (QIAGEN) and sequenced to confirm virus species. RNA from samples positive for coronavirus was then used for cDNA synthesis by using random hexamer, gene-specific, and oligo(dT) primers. The RdRp gene and 1b open reading frames, including the helicase (HEL) domain, were then sequenced, also as previously described (*4*). Sequences derived from this study were deposited in GenBank (accession nos. EU769557 and EU769558).

**Figure 1:**
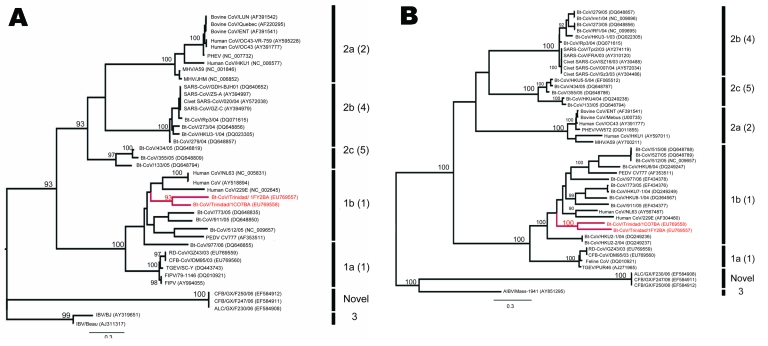
Maximum likelihood trees of coronaviruses based on A) 780-bp fragment of the RNA-dependent RNA polymerase gene and B) 1,797 bp of the helicase (HEL) domain of open reading frame 1b. Trees were inferred under the General Time Reversible (GTR + Γ_4_ + I) model by using PAUP* version 4.0b (Sinauer Associates, Inc., Sunderland, MA, USA). Bootstrap support values >90% are indicated. Previously defined phylogenetic groups and a putative novel group (*10*) are delineated by the bars on the right. The numbering of these groups is as described in the eighth report of the International Committee on Taxonomy of Viruses with the alternative grouping proposed by Tang et al*.* (*4*) in brackets. Trinidadian bat coronavirus sequences are highlighted in red. GenBank accession numbers are noted in parentheses. Scale bars indicate number of nucleotide substitutions per site.

**Figure 2:**
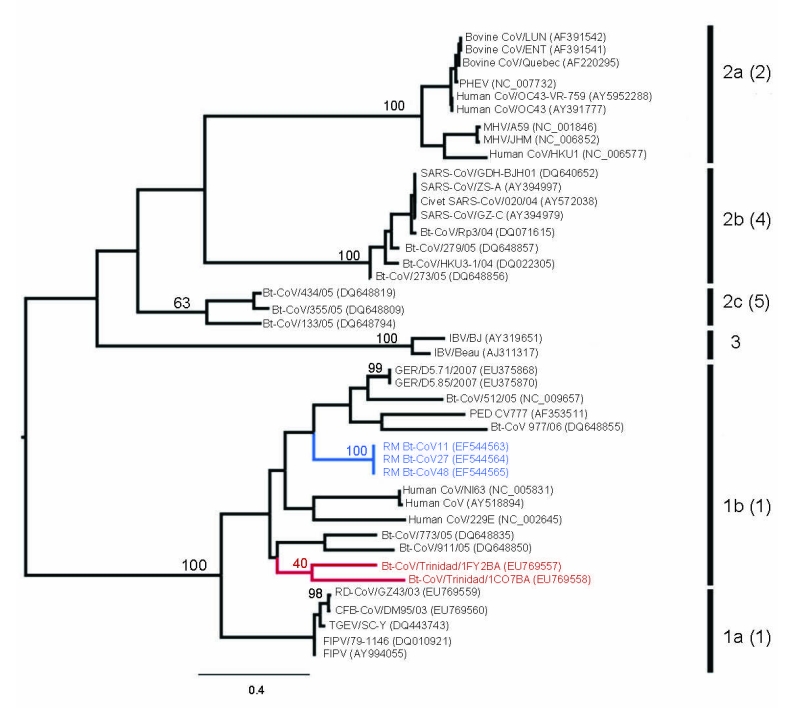
Maximum likelihood tree of coronaviruses based on 378-bp fragment of the RNA-dependent RNA polymerase gene. The tree was inferred under the General Time Reversible (GTR + Γ_4_ + I) by using PAUP* version 4.0b (Sinauer Associates, Inc., Sunderland, MA, USA). Trinidadian bat coronavirus (Bt-CoV) sequences are highlighted in red and North American Bt-CoV in blue. Previously defined phylogenetic groups and a putative novel group (*10*) are delineated by the bars on the right. The numbering of these groups is as described in the eighth report of the International Committee on Taxonomy of Viruses with the alternative grouping proposed by Tang et al*.* (*4*) in brackets. Bootstrap support values for groups 1a, 1b, 2a–c, 3, and the lineage containing Trinidadian Bt-CoVs are shown. GenBank accession numbers are noted in parentheses. Scale bar indicates number of nucleotide substitutions per site.

Sequences were aligned with previously published CoV sequences from GenBank by using ClustalX (http://bips.u-strasbg.fr/fr/Documentation/ClustalX) then manually aligned by using the Se-Al program (http://tree.bio.ed.ac.uk/software/seal). The GenBank accession numbers of all sequences used are noted in the taxon names in Figures 1 and 2. The RdRp sequences were trimmed to equal length, which created 2 datasets of 780 bp (n = 40) and 378 bp (n = 45). The latter included Bt-CoV sequences from North America and Germany that were too short to be included in the first dataset. A third dataset comprised an alignment of the HEL domain (n = 46) trimmed to 1,797 bp. Maximum likelihood (ML) phylogenies were inferred under a General Time Reversible (GTR + Γ_4_ + I) model, which was identified as the best-fit model of nucleotide substitution using MODELTEST version 3.7 (*10*). Bootstrapping was performed to assess the robustness of tree topologies by using 1,000 replicate neighbor-joining (NJ) trees under the ML substitution model. All analyses were performed with PAUP* version 4.0b (Sinauer Associates, Inc., Sunderland, MA, USA).

CoV RNA was detected in 1 of 21 *Glossophaga* *soricina* and 1 of 5 *Carollia* *perspicillata* bats tested**.** The latter, designated Bt-CoV/Trinidad/1FY2B, was an adult male from the area of Fyzabad, and virus was detected in anal and oropharyngeal swabs. The infected *G.* *soricina* specimen (Bt-CoV/Trinidad/1CO7B) was an adult female from the area of Couva, and the virus was detected in the anal swab only. Subsequent sequencing was performed on virus from the anal swabs. A total of 3,905 bp of RdRp from Bt-CoV/Tri/1CO7B and 5,160 bp from Bt-CoV/Tri/1FY2B were sequenced, in addition to 1,782 bp from the HEL domain of both samples (full sequence data not shown). Across the shared 3,798-bp region of RdRp, divergence was 28.7% at the nucleotide level and 15.5% at the amino acid level. Similarly, the HEL domains of the 2 viruses showed 27.3% divergence at the nucleotide level and 13.3% at the amino acid level.

ML phylogenies inferred from the RdRp genes of 40 viruses (780 bp) and the HEL domains of 46 viruses (1,797 bp) are shown in Figure 1. In both cases, the 5 groups proposed by Tang et al. (*4*) on the basis of NJ trees, and the lineage containing the recently reported novel CoV sequences (*11*), were strongly supported with bootstrap values >95% in all cases. In each case the Trinidadian sequences clustered with group 1 CoVs within a clade containing all other group 1 bat and human CoVs as well as porcine CoV. To determine the phylogenetic relationship between the Trinidadian Bt-CoVs and North American Bt-CoVs, for which only relatively short sequences for RdRp were available, we inferred a second RdRp ML phylogeny based on a 378-bp fragment (Figure 2). When this shorter fragment was used, 5 groups were again defined, but bootstrap support for the putative group 5 (*4*) was lower. The level of divergence between Trinidadian sequences was notably higher than among the North American (*6*) and German sequences (*7*).

## Conclusions

Our detection of RNA from group 1 CoVs in Trinidadian bats shows that Bt-CoVs have a wider distribution than previously suspected and is added support for bats as the original host species for these viruses. Group 1 CoVs form 2 well-supported clades designated 1a and 1b (*12*). The Trinidadian bt-CoV clustered within the latter clade, which contains all other group 1 Bt-CoVs, including those from Germany and North America, and the 3 known group 1 human CoVs associated with respiratory illness (*13*–*15*). Despite the geographic proximity of the bats from which the Trinidadian Bt-CoV sequences were derived—Couva and Fyzabad are 28 km (17 miles) apart, and Trinidad has an area of only 4,769 km^2^ (1,864 square miles)—they are relatively highly divergent. This divergence might reflect virus adaptations to different host species; however, more data would be needed to confirm this. Given the mobility of bats, the possibility of the viruses having different geographic origins (perhaps even from outside Trinidad) cannot be ruled out. Further work on CoV diversity in Trinidad and the rest of the Americas, as well as on the ecology and behavior of susceptible bat species, is needed to understand the origins, evolution, and dispersal of these viruses.
